# Donor-Derived Cell-Free DNA as a Companion Biomarker for AMR Treatment With Daratumumab: Case Series

**DOI:** 10.3389/ti.2024.13213

**Published:** 2024-08-01

**Authors:** Bilgin Osmanodja, Aylin Akifova, Klemens Budde, Michael Oellerich, Julia Beck, Kirsten Bornemann-Kolatzki, Ekkehard Schütz, Joachim Velden, Claudia Lehmann, Bastian Malte Krüger, Anette Bachmann, Jan Kowald

**Affiliations:** ^1^ Department of Nephrology and Intensive Care, Charité—Universitätsmedizin Berlin, Corporate Member of Freie Universität Berlin, Berlin Institute of Health, Humboldt-Universität zu Berlin, Berlin, Germany; ^2^ Department of Clinical Pharmacology, University Medical Center Göttingen, Göttingen, Germany; ^3^ Chronix Biomedical GmbH, Göttingen, Germany; ^4^ Department of Nephropathology, Institute of Pathology, University Hospital Erlangen, Friedrich-Alexander University Erlangen-Nuremberg, Erlangen, Germany; ^5^ Institute for Transfusion Medicine, Laboratory for Transplantation Immunology, University Hospital Leipzig, Leipzig, Germany; ^6^ Medical Department III, Division of Nephrology, University of Leipzig Medical Center, Leipzig, Germany

**Keywords:** kidney transplantation, antibody-mediated rejection, daratumumab, donor-derived cell-free DNA, biomarker

## Abstract

Antibody-mediated rejection (AMR) is among the most frequent causes for graft loss after kidney transplantation. While there are no approved therapies, several case reports with daratumumab and the very recent phase 2 trial of felzartamab in AMR have indicated the potential efficacy of therapeutic interventions targeting CD38. Donor-derived cell-free DNA (dd-cfDNA) is an emerging biomarker with injury-specific release and a short half-life, which could facilitate early diagnosis of AMR and monitoring of treatment response. We describe two cases of patients with chronic active AMR, who were treated with monthly daratumumab infusions, and in whom donor-derived cell-free DNA (dd-cfDNA) was measured longitudinally to monitor treatment response. In both patients, daratumumab treatment led to stabilization of kidney function parameters, a strong decline of dd-cfDNA below the previously established threshold for rejection, and partial or complete histologic resolution of AMR activity. Our case series suggests that dd-cfDNA may be a useful companion biomarker for longitudinal monitoring of anti-CD38 treatment in patients with AMR.

## Graphical Abstract

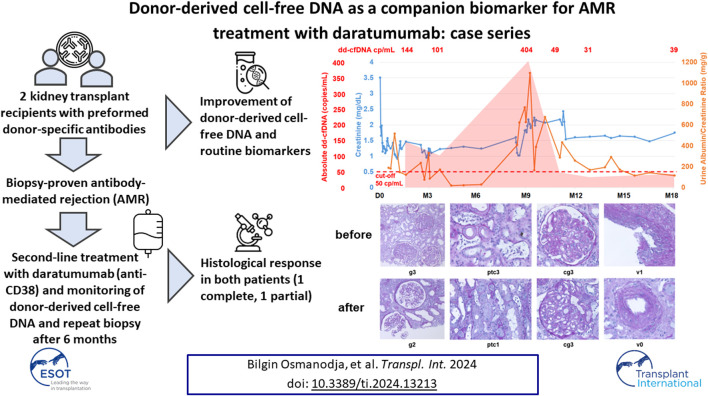

## Introduction

Antibody-mediated rejection (AMR) is among the most frequent causes for graft loss after kidney transplantation [1]. Treatment of AMR remains a challenge, and while there are no approved therapies [2–7], several case reports have indicated the potential efficacy of therapeutic interventions targeting CD38 [8–11]. Recently, a phase 2 trial of felzartamab, an investigational, fully human IgG1 monoclonal anti-CD38 antibody in patients with AMR demonstrated safety and tolerability, and showed resolution of AMR in a majority of patients [12]. The proposed mechanisms of action of anti-CD38 treatment are depletion of alloantibody-producing plasma cells (PC) and natural killer (NK) cells, the latter of which are key effector cells in the pathogenesis of AMR [13].

Meanwhile, off-label use of daratumumab has been performed, which is currently approved for multiple myeloma. Clinical routine parameters such as creatinine or estimated glomerular filtration rate (eGFR) and urine albumin-creatinine ratio (uACR) are neither optimal to monitor AMR activity nor treatment response. Donor-derived cell-free DNA (dd-cfDNA) is an emerging biomarker with injury-specific release and a short half-life, which could facilitate early diagnosis of AMR and monitoring of treatment response [14].

In this case series, we demonstrate the use of dd-cfDNA for longitudinal graft monitoring in two patients with AMR that were treated with daratumumab as second line therapy.

## Methods

### Daratumumab Treatment

Daratumumab was administered as an intravenous infusion in a dosage of 16 mg/kg body weight, every 4 weeks. The first infusion was started at a rate of 25 mL/h and was subsequently increased by 25 mL/h every 30 min up to a maximum of 200 mL/h. Subsequent infusions were started at 50 mL/h and increased by 50 mL/h every 30 min up to a maximum rate of 200 mL/h.

Premedication included prednisolone (100 mg), dimetindene (4 mg), cimetidine (200 mg), ondansetron (4 mg), paracetamol (1,000 mg) and montelukast (10 mg).

Since daratumumab is associated with increased risk of opportunistic infections, both patients received pneumocystis prophylaxis and, due to intermediate risk of cytomegalovirus infection, antiviral prophylaxis with valganciclovir [15].

### Donor-Derived Cell-Free DNA Testing

Measurement of dd-cfDNA was performed as described previously [16, 17]. In brief, for each patient, four informative single-nucleotide polymorphisms (SNPs), defined as an SNP for which the recipient has a homozygous allelic state, and the graft carries at least one heterozygous allele, were selected from a predefined set of 40 SNPs. These four SNPs were used to quantify the dd-cfDNA (%) concentration, which is defined as donor-alleles/(donor-alleles + recipient-alleles). Results for SNPs with heterozygous graft genotypes were corrected by a factor of two. Total cfDNA was extracted from up to 8 mL plasma collected in certified blood collection tubes (Streck Corp., Omaha, NE, United States). The concentration was determined using droplet-digital PCR (ddPCR) and was corrected for extraction loss and cfDNA fragmentation as described previously [16]. The absolute concentration of dd-cfDNA per mL plasma was calculated by multiplying total cfDNA (copies/mL) and dd-cfDNA (%). An abnormal dd-cfDNA result was defined as a value of >50 copies/mL for absolute quantification [16, 17].

### Detection and Differentiation of HLA Antibodies With Bead-Based Technique

HLA-antibody differentiation was performed using Luminex Single Antigen Bead assays (One Lambda, West Hills, CA, United States) LSA1A04 and LSA2A01. The assays have been performed according to the manufacturer’s instructions. The antibody determination was performed on the Luminex 200 device using xPONENT^®^ software and was analyzed in the HLA-Fusion software v4.4. MFI values > 1,500 were defined as the cut-off for positive detection of HLA antibodies in the single tests.

### Statement of Ethics

Written informed consent was obtained from both patients for publication of the details of their medical case and any accompanying images. The underlying observational study involving human participants were reviewed and approved by the ethics committee of Charité - Universitätsmedizin Berlin (EA2/144/20, date of approval 25.02.2021). The patients provided written informed consent to participate in this study. The clinical activities being reported are consistent with the principles of the Declaration of Istanbul as outlined in the “Declaration of Istanbul on Organ Trafficking and Transplant Tourism”.

## Results

### Case Descriptions

#### Case 1

The first patient is a 52-year-old female with autosomal dominant polycystic kidney disease (ADPKD), who received a living-donor kidney from her husband despite preformed donor-specific anti-HLA antibodies (DSA) against donor antigen DR7 (mean fluorescence intensity [MFI] 3339) triggered by a previous pregnancy. The donor-recipient HLA-A, -B, -C, -DR, -DQ mismatch grade was 10 (2-2-2-2-2).

**FIGURE 1:**
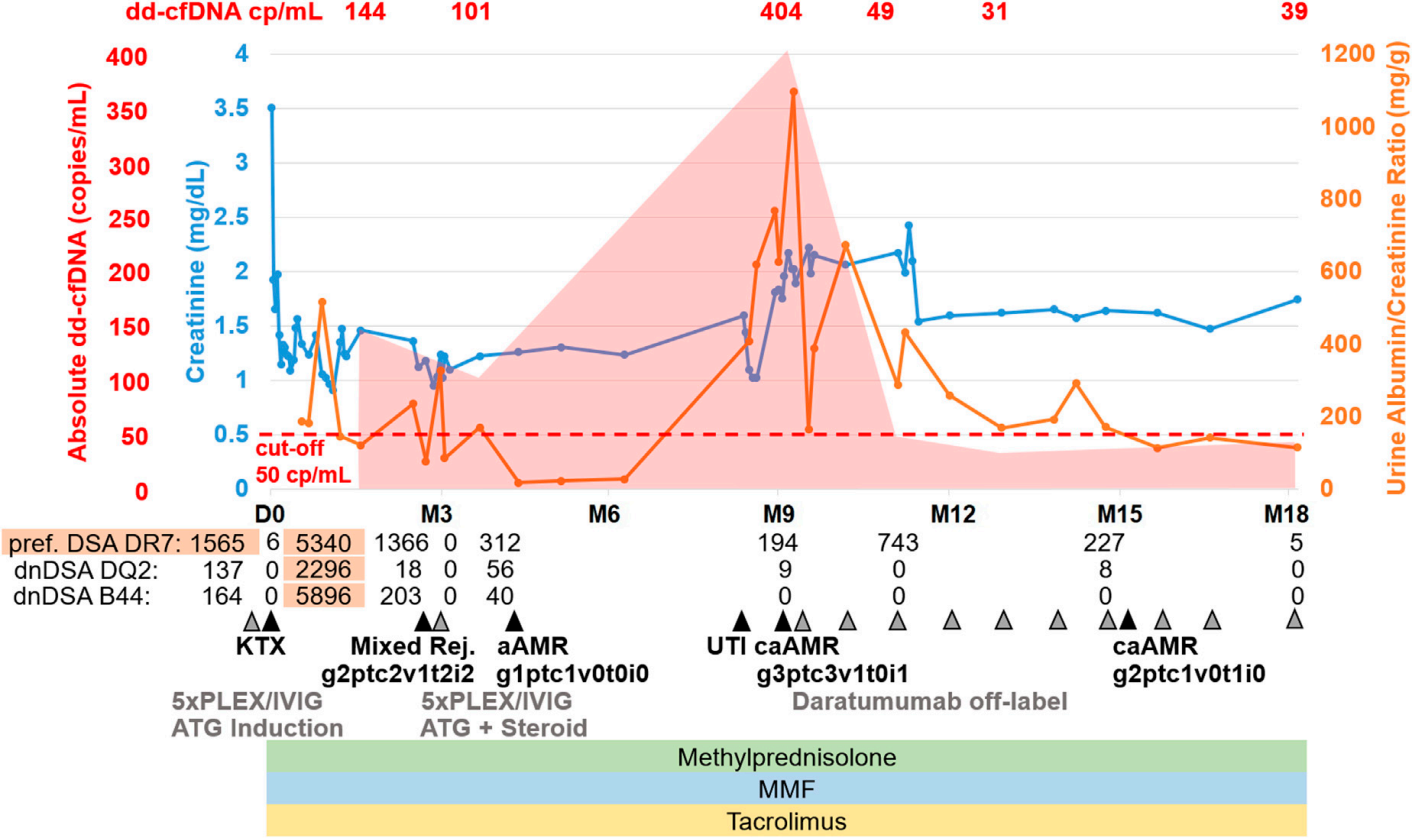
Case summary including key clinical events, allograft biopsy results, therapeutic interventions, routine biomarkers (creatinine, urine albumin/creatinine ratio) as well as donor-derived cell-free DNA (dd-cfDNA). The red shaded area depicts the absolute dd-cfDNA levels and numeric values are provided at the top of the figure. The prespecified cut-off of 50 copies/mL derived from previous validation studies is shown. Grey arrowheads indicate therapeutic interventions, black arrowheads indicate clinical events including kidney allograft biopsies. Orange shape indicate the time periods when DSA MFI were above 1,500. dd-cfDNA, donor-derived cell-free DNA; cp, copies; D0, day of transplantation; M, months after transplantation; DSA, donor-specific anti-HLA antibodies; dnDSA, *de novo* DSA; KTX, kidney transplantation; PLEX, plasma exchange; IVIG, intravenous immunoglobulins; ATG, anti-thymocyte globulin; Mixed Rej., mixed rejection; aAMR, active antibody-mediated rejection; TCMR, T-cell mediated rejection; UTI, urinary tract infection; caAMR, chronic active antibody-mediated rejection; MMF, mycophenolate mofetil.

Before kidney transplantation, desensitization with 5 cycles of plasma exchange (PLEX) and intravenous immunoglobulins (IVIG) was performed per institutional standard. Preformed DSA declined to an MFI of 1,564, which was deemed sufficiently low to perform transplantation (Figure 1). Two weeks after kidney transplantation and induction immunosuppression with anti-thymocyte globulin (ATG; three doses of 1.25 mg/kg each), as well as triple maintenance immunosuppression with methylprednisolone, tacrolimus (through levels 10–12 ng/mL) and mycophenolate mofetil (MMF; 2 g/d), the preformed DSA MFI increased to 5339, and *de novo* DSA (dnDSA) against DQ2 (MFI 2296) and B44 (MFI 5896) occurred.

Six weeks after transplantation, we observed a rise in creatinine from a best value of 1.0 mg/dL to 1.5 mg/dL without a significant increase in urine albumin/creatinine ratio (uACR). At that time, DSA MFI have already declined below 1,500 (DR7 1366, DQ2 18, B44 203). We performed donor-derived cell-free DNA testing, which showed markedly increased levels of 144 copies/mL (2.61%) compared with the prespecified cut-offs of 50 copies/mL and 0.5%. Given the history of the patient, this was highly suggestive of AMR. We performed kidney biopsy, which confirmed active antibody-mediated rejection (aAMR) and showed acute T-cell mediated rejection (TCMR) Banff IIA (g2ptc2v1t2i2tIFTA1iIFTA0cg0mm0cv1aah0ct1ci0).

The patient was treated with 5 PLEX and IVIG (10 g after each PLEX) as well as ATG (three doses of 1.25 mg/kg each) and steroid pulse for TCMR. Tacrolimus levels were maintained between 6–8 ng/mL and MMF dose was 2 g/d. Kidney function parameters stabilized, but dd-cfDNA was still increased with 101 copies/mL (0.84%), suggesting ongoing graft injury. However, due to the clinical improvement of kidney function (serum creatinine 1.1 mg/dL), we chose not to further intensify the immunosuppressive regimen. The day after completion of PLEX, MFI for all DSA were 0; 6 weeks after PLEX, MFI were still low for all DSA (DR7 312, DQ2 56, B44 40). A second biopsy 2 months after the initial biopsy showed complete resolution of TCMR and partial improvement of AMR activity (g1ptc1v0t0i0ti2tIFTA0iIFTA3cg0mm0cv2aah1ct2ci2).

**FIGURE 2:**
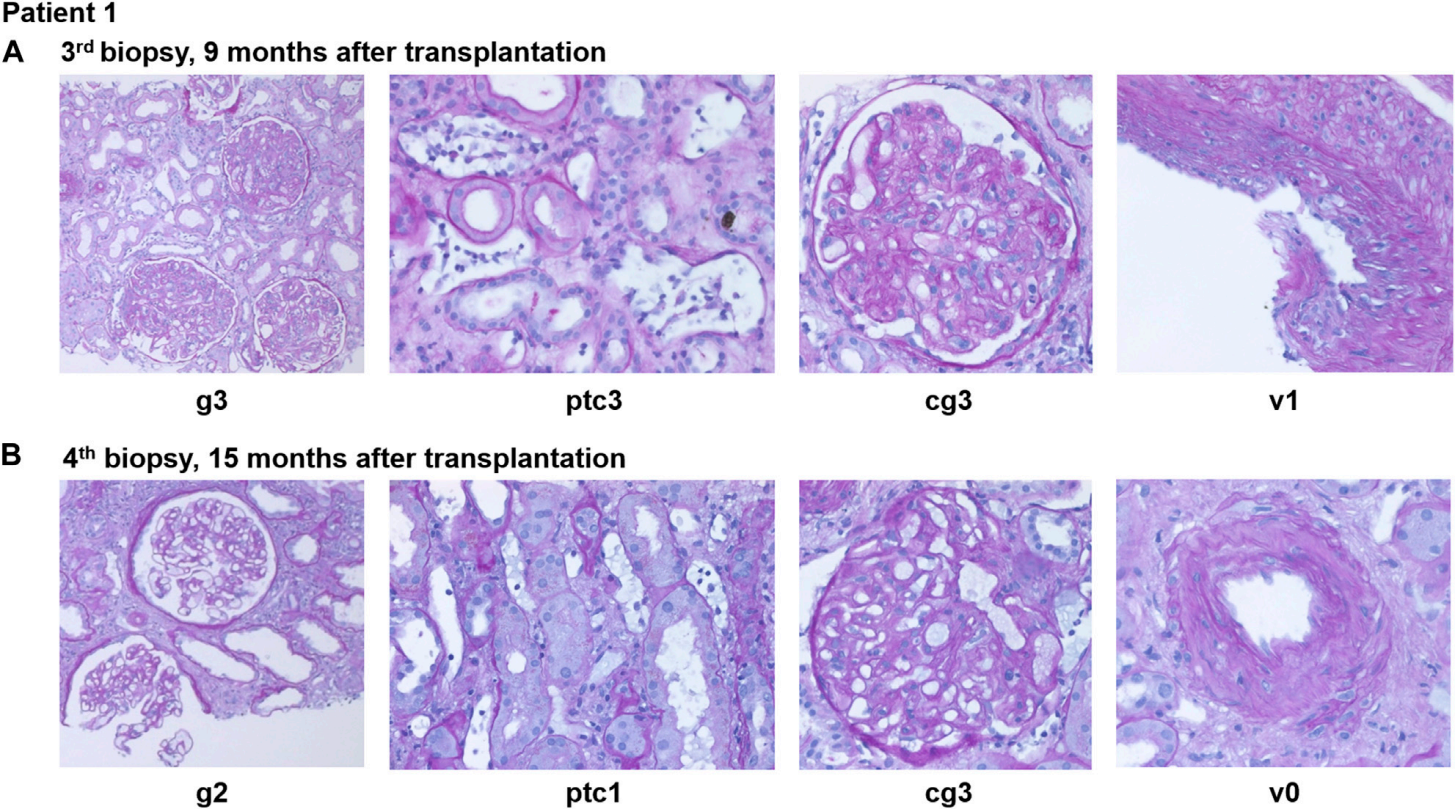
Representative histopathological findings for patient 1 showing glomerulitis (g), peritubular capillaritis (ptc), chronic transplant glomerulopathy (cg), and intimal arteritis (v) at **(A)** 9 months after transplantation (before daratumumab) and **(B)** 15 months after transplantation (6 months after treatment with daratumumab).

Six months later, the patient had a severe urinary tract infection (UTI) and a subsequent deterioration of kidney function with increases in serum creatinine and uACR. Two weeks after successful treatment of the UTI (no leukocyturia, normal CRP), we performed dd-cfDNA testing, which showed very high levels of 404 copies/mL (0.7%). DSA MFI at this point had been stable since PLEX (DR7 194, DQ2 9, B44 0). A third kidney biopsy confirmed ongoing chronic active AMR (caAMR) with severe transplant glomerulopathy (cg3), severe glomerulitis (g3), severe peritubular capillaritis (ptc3), mild to moderate intimal arteritis (v1), matching the high dd-cfDNA levels (Figure 2A) (g3ptc3v1t0i1ti1tIFTA0iIFTA0cg3mm0cv1aah3ct1ci1). Since there was no approved treatment available, repeated DSA elimination was not promising due to low MFI, and some recent case reports suggested potential efficacy, we decided to perform off-label treatment with daratumumab as outlined above with regular monitoring of kidney function parameters, DSA, dd-cfDNA and repeat biopsy after 6 months. Maintenance immunosuppression with tacrolimus (through levels 6–8 ng/mL) and methylprednisolone (4 mg/d) was continued, but MMF dose was reduced (1 g/d) due to leukopenia (2,800/µL) 1 week after first administration of daratumumab.

Kidney function stabilized after initiation of treatment and donor-derived cell-free DNA normalized 8 weeks after starting daratumumab (49 copies/mL; 0.37%) and further decreased at 16 weeks (31 copies/mL; 0.22%) and 9 months (39 copies/mL; 0.25%). Three months after treatment initiation, creatinine was around 1.5 mg/dL and uACR below 300 mg/g and MFI <1,500 (DR7 680 and DQ2 34).

Peripheral blood NK cell count declined shortly after initiation of daratumumab from 120/nL to less than 20/nL and stabilized at 11-12/nL after 4 months of treatment.

A fourth biopsy after 6 months still showed caAMR with unchanged chronic changes (cg3cv1ci1ct1), but improved Banff scores for AMR activity: glomerulitis decreased from severe (g3) to moderate (g2), and peritubular capillaritis improved from severe (ptc3) to mild (ptc1), while the intimal arteritis (v1) was no longer detectable (v0), Figure 2B (g2ptc1v0t1i0ti1tIFTA0iIFTA3cg3mm0cv1aah2ct1ci1).

Given the good response, we extended therapy with daratumumab to 9 months, and increased the interval to 6 weeks with repeated monitoring of laboratory values.

During the initial administration of daratumumab, nausea and vomiting occurred, which disappeared after administration of antiemetic therapy. Other infusion-related reactions, in particular bronchospasm or shortness of breath, did not occur with the premedication including montelukast.

Two months after initiation of daratumumab, the patient developed SARS-CoV-2-infection. She was admitted to hospital and treated with remdesivir for 3 days, but only mild symptoms were observed. In this context, severe hypogammaglobulinemia (serum-IgG-level <4 g/L) was detected. A single dose of 30 g IVIG was administered. Subsequently, only mild hypogammaglobulinemia was detected (serum-IgG-level >4 g/L). There were no further hospitalizations.

The main complications during daratumumab treatment were recurrent UTIs treated with oral antibiotics and not requiring hospitalization. Eventually, the patient was started on antibiotic prophylaxis with nitroxoline after which no further UTIs were observed. As the UTIs had already occurred before starting daratumumab treatment, they were not necessarily treatment-related.

At the last visit, the patient is in good condition with serum creatinine of 1.5 mg/dL, uACR 114 mg/g, MFI practically undetectable (DR7 5, DQ2 0, and B44 0), and dd-cfDNA of 39 copies/mL (0.25%), while blood pressure medication was partially discontinued.

#### Case 2

The second patient is a 25-year-old female patient with Mayer-Rokitanski-Küster-Hauser syndrome, who underwent a second kidney transplantation at the age of 24 within the Eurotransplant Acceptable Mismatch program (vPRA 97.2%). After a 1-year period on peritoneal dialysis at the age of two, she underwent first kidney transplantation at the age of three. Due to early urological complications with recurrent UTIs, an ileal conduit urinary diversion was performed. Eventually, the graft was functioning for 18.5 years. Afterwards, the patient was on hemodialysis for almost 4 years before the second transplantation was performed.

**FIGURE 3:**
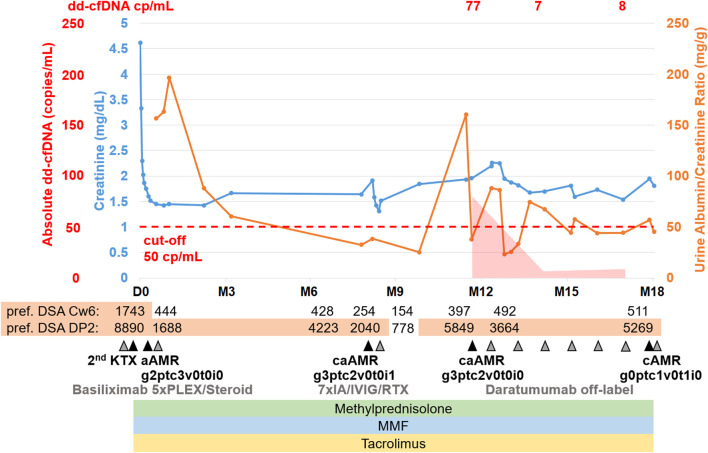
Case summary including key clinical events, allograft biopsy results, therapeutic interventions, routine biomarkers (creatinine, urine albumin/creatinine ratio) as well as donor-derived cell-free DNA (dd-cfDNA). The red shaded area depicts the absolute dd-cfDNA levels and numeric values are provided at the top of the figure. The prespecified cut-off of 50 copies/mL derived from previous validation studies is shown. Grey arrowheads indicate therapeutic interventions, black arrowheads indicate clinical events including kidney allograft biopsies. Orange shape indicate the time periods when DSA MFI were above 1,500. dd-cfDNA, donor-derived cell-free DNA; cp, copies; D0, day of transplantation; M, months after transplantation; DSA, donor-specific anti-HLA antibodies; KTX, kidney transplantation; PLEX, plasma exchange; IVIG, intravenous immunoglobulins; RTX, rituximab; aAMR, active antibody-mediated rejection; cAMR, chronic inactive antibody-mediated rejection; MMF, mycophenolate mofetil.

Despite well matched graft (mismatch grade HLA-A, -B, -C, -DR, -DQ 1-1-1-0-0) within the Acceptable Mismatch (AM) program, two preformed DSA were present (Cw6; MFI 1743, DP2; MFI 8890) after transplantation (Figure 3). Initial immunosuppression consisted of Interleukin-2 receptor antibody (basiliximab), tacrolimus (through levels 10–12 ng/mL), MMF (2 g/d) and methylprednisolone. Due to delayed graft function, a first kidney biopsy was performed on day 9 after transplantation and showed active AMR (g2ptc3v0i0t0ti0tIFTA0iIFTA0cg0mm0cv0aah0ct0ci0). A steroid pulse and 5 courses of PLEX were initiated after which creatinine improved to a baseline of 1.5 mg/dL with low uACR. While the preformed DSA against Cw6 were decreasing to MFI <1,500 (444 after PLEX), the antibodies against DP2 persisted at MFI 1688 after PLEX. Clinical routine parameters remained stable; therefore, no additional anti-rejection therapy was initiated and maintenance immunosuppression with tacrolimus (through levels 10–12 ng/mL), MMF (2 g/d) and methylprednisolone (5 mg/d) was continued. A follow-up biopsy was declined by the patient due to stabilization of kidney function. Seven months after transplantation, DSA MFI of DP2 increased to 4,223, and DSA MFI of Cw6 remained stable below the cutoff at 428.

**FIGURE 4:**
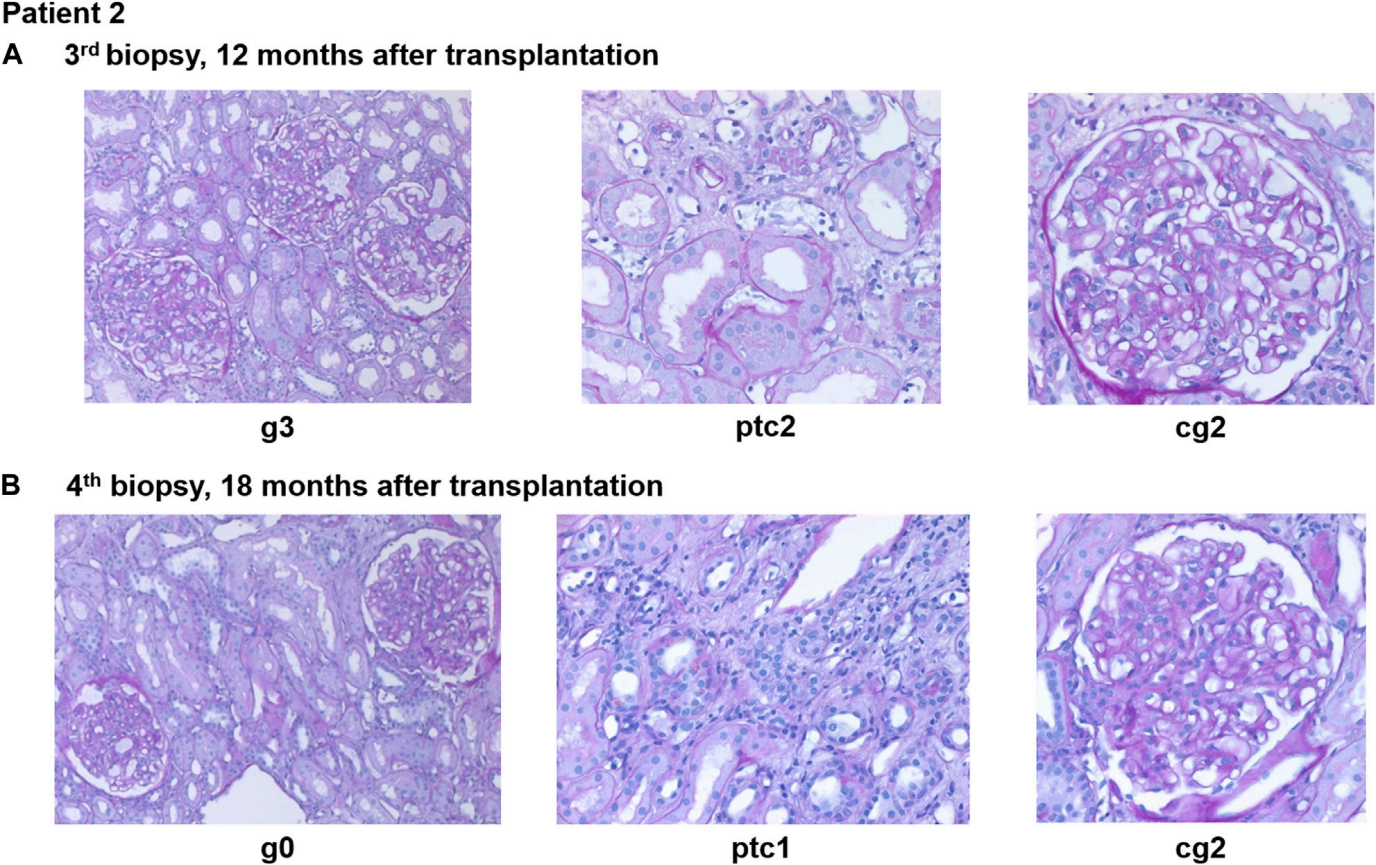
Representative histopathological findings for patient 2 showing glomerulitis (g), peritubular capillaritis (ptc), and chronic transplant glomerulopathy (cg) at **(A)** 12 months after transplantation (before daratumumab) and **(B)** 18 months after transplantation (6 months after treatment with daratumumab).

Eight months after transplantation, a rise in creatinine (1.6–1.9 mg/dL) was observed, while DSA MFI were decreasing (DP2 2040, Cw6 241). A second biopsy showed caAMR (g3ptc2v0t0i1ti1tIFTA0iIFTA0cg3mm0cv0aah0ct1ci1). Due to persistent DSA, a first-line therapy with immunoadsorption (IA), IVIG (10 g after each IA) and rituximab (RTX; 375 mg/m^2^ body surface) was initiated and led to a transient decrease in creatinine and effectively lowered DSA for 2 months (DP2 778, Cw6 155). However, 2-3 months later, creatinine rose again (1.9–2.3 mg/dL), and a third biopsy still showed caAMR with high activity (g3ptc2v0t0i0ti0tIFTA0iIFTA0cg2mm0cv0aah0ct1ci1, Figure 4A). In line with the histology, dd-cfDNA was elevated at the time of biopsy (77 cp/mL; 0.55%). MFI of DP2 DSA increased to 5849 (Cw6 397).

Again, we decided to perform off-label treatment with daratumumab with regular monitoring of kidney function parameters, dd-cfDNA and repeat biopsy after 6 months. Similar to the first patient, this patient also developed nausea and vomiting during the first administration of daratumumab. No other infusion-related reactions occurred in this patient. Only mild hypogammaglobulinemia was detected (serum-IgG-level 5.3 g/L) after 2 months of treatment. There were no infectious complications and no hospitalizations. During the first 3 months of daratumumab treatment, leukopenia (minimum 2,100/µL) was detected 1 week after daratumumab administration. MMF dose was reduced to 1 g/d, while tacrolimus (through levels 6–8 ng/mL) and methylprednisolone (4 mg/d) remained unchanged.

After 2 months, creatinine stabilized at 1.7 mg/dL and uACR was below 100 mg/g. Dd-cfDNA declined to the lower limit of detection (7 copies/mL; 0.1%) after 2 months of treatment and was still low after 5 months (8 copies/mL; 0.28%).

NK cell count declined after initiation of daratumumab from 53/nL to 20/nL after 3 months of treatment and 18/nL after 4 months of treatment.

A fourth biopsy after 6 months of daratumumab therapy showed chronic (inactive) AMR with AMR activity being almost completely resolved (g0ptc1v0t1i0ti1iIFTA2tIFTA1cg2mm1cv1aah2ct1ci1), which is matching the dd-cfDNA results (Figure 4B). The therapy with daratumumab will be extended to 9 months and time intervals will be increased under close monitoring.

## Discussion

This is the first case series to demonstrate the use of dd-cfDNA to monitor treatment response to anti-CD38 therapy with daratumumab in two sensitized patients with AMR after kidney transplantation.

Histologically, we observed partial response of AMR activity in the first patient, and virtually complete resolution of AMR activity in the second one. After treatment initiation with daratumumab, the injury biomarker dd-cfDNA showed a strong decline below the previously established threshold for rejection in both patients. However, in the patient with remaining AMR activity after 6 months of treatment, dd-cfDNA remained higher than the median of 25 copies/mL that was established in stable phase patients in the original validation study, while the second patient showed dd-cfDNA at the lower limit of detection [16].

In both patients, serum creatinine levels eventually stabilized and uACR declined, which - together with histopathological parameters and dd-cfDNA course - we interpreted as clinically meaningful treatment response.

Clinical routine parameters showed a slower and a less pronounced response to treatment initiation than dd-cfDNA. This is due to chronic damage in both patients and the general disadvantages of creatinine and uACR as markers of injury. While uACR responded faster after treatment initiation than creatinine, chronic transplant glomerulopathy may represent chronic structural damage and can lead to persistent albuminuria, which hence does not necessarily reflect AMR activity.

Given the natural course of caAMR with progressive worsening of kidney function, this anecdotal evidence still suggests a meaningful therapeutic effect of daratumumab. Unfortunately, both patients had already developed mild to moderate chronic lesions (cg2-3, ci1, ct1, and cv0-1) at initiation of treatment, which are known to indicate limited long-term success [18].

Recently, a phase 2 trial of felzartamab in AMR demonstrated acceptable safety and tolerability, and promising results with 81.8% of patients showing complete histological resolution of AMR after 24 weeks of treatment as compared to 20% in the placebo group as well as improvement in microvascular inflammation in normal histology, AMR-related transcripts, and dd-cfDNA as marker of injury [12].

While daratumumab is the first fully human anti-CD38 monoclonal antibody, and induces complement-dependent cytotoxicity (CDC), antibody-dependent cell-mediated cytotoxicity (ADCC), as well as antibody-dependent cellular phagocytosis (ADCP), felzartamab has a λ instead of κ light chain, and only induces ADCC and ADCP [19]. The third anti-CD38 monoclonal antibody available, isatuximab, additionally induces direct apoptosis of plasma cells. Different effects on the bone marrow microenvironment have been discussed in multiple myeloma, but the clinical relevance is not yet understood in the context of AMR [19, 20].

Practically, felzartamab and daratumumab are usually given as an induction therapy (e.g., 4 weekly doses for felzartamab), followed by maintenance therapy (e.g., 5 monthly doses for felzartamab), which is derived from the treatment of multiple myeloma [12, 21]. For the feasibility of outpatient treatment, we initiated daratumumab as 4-weekly infusions without an induction period with weekly infusions, discordant to previously established regimens for multiple myeloma and to the protocol used in the felzartamab trial [12]. Both patients developed nausea during first dose administration, but the absence of a severe first dose reaction could be due to the extensive premedication and slow initial infusion rate (25 mL/h). In the felzartamab trial, the rate of infusion-related reactions in the felzartamab group was 72.7% [12].

Even without an induction period, we observed a pronounced and persistent reduction of NK cells in both patients, which was comparable to the one seen in the felzartamab trial [12]. While we did not assess plasma cell depletion in bone marrow biopsies, the vanishing AMR activity in the presence of detectable DSA in the second patient suggests that depletion of NK cells instead of antibody-producing plasma cells is the mechanism of action.

Accordingly, the felzartamab trial showed depletion of NK cells, but only moderate reduction in DSA MFI, which led the authors to similar conclusions [12]. In line with this, it has been shown that NK cell-mediated endothelial cytotoxicity plays an important role in DSA-negative recipients with histological evidence of microvascular inflammation [22]. If this hypothesis holds true, monitoring peripheral blood NK cell count and assessing treatment response with dd-cfDNA could enable personalized dosing intervals and treatment regimens, which has also been proposed by the authors of the felzartamab trial [12].

Future studies need to evaluate whether an induction period as established for myeloma is necessary for AMR treatment, especially if NK cell depletion can be confirmed to be the main mechanism of action. Furthermore, subcutaneous instead of intravenous application of daratumumab is more feasible and was noninferior in patients with multiple myeloma but showed an improved safety profile and should be considered depending on the reimbursement and local availability [21].

In the felzartamab trial, there was a high recurrence rate of 1/3 in the initial felzartamab responders at week 52, after treatment has been stopped for 6 months. In light of these results, we decided to continue treatment with increasing dosing intervals under close monitoring of dd-cfDNA and NK cell count, as well as routine biomarkers [12].

Nevertheless, both cases also demonstrate that sensitized patients undergoing transplantation against preformed DSA are at high-risk for AMR and potentially inferior graft survival and that currently used desensitization regimens are insufficient to prevent AMR. Therefore, rigorous patient information and selection is necessary to avoid complications and the need for intensified immunosuppressive regimens. If desensitization remains the only option, adding daratumumab or another anti-CD38 agent to novel regimens may reduce the risk of AMR and improve clinical outcomes [23].

In summary, we suggest that dd-cfDNA may be a useful companion biomarker for longitudinal monitoring of anti-CD38 treatment in patients with AMR.

## Data Availability

The original contributions presented in the study are included in the article/supplementary material, further inquiries can be directed to the corresponding author.
